# (3*S*,4*R*,4a*S*,7a*R*,12b*S*)-3-Cyclo­propyl­meth­yl-4a,9-dihy­droxy-3-methyl-7-oxo-2,3,4,4a,5,6,7,7a-octa­hydro-1*H*-4,12-methano-1-benzofuro[3,2-*e*]isoquinolin-3-ium 2,2,2-trifluoro­acetate methanol solvate

**DOI:** 10.1107/S1600536810041164

**Published:** 2010-10-20

**Authors:** Xu Cai, Xinbo Zhou, Zhibing Zheng, Wu Zhong, Song Li

**Affiliations:** a Beijing Institute of Pharmacology and Toxicology, Beijing, 100850, People’s Republic of China

## Abstract

In the title compound, C_21_H_26_F_3_NO_6_^+^·CF_3_COO^−^·CH_3_OH or *S*-MNTX·CF_3_COO^−^·CH_3_OH (MNTX = methyl­naltrexone), the conformation of the polycyclic backbone of the noroxy­morphone skeleton can be simplified in terms of the angles between the least-squares planes of these rings. The dihedral angle between the cyclohexene and piperidine rings is 84.5 (6)°, while the dihedral angles between the planes of cyclohexane ring and the benzene, cyclohexene and piperidine rings, respectively, are 85.8 (6),80.0  (7) and 10.3 (7)°. In the crystal, mol­ecules are linked by O—H⋯O hydrogen bonds. The trifluoro­acetate F atoms are disordered in a 0.710 (14):0.710 (14) ratio. The absolute stereochemistry was inferred from the use of (4*R*,4a*S*,7a*R*,12b*S*)-3-(cyclo­propyl­meth­yl)-4a,9-dihy­droxy-2,3,4,4a,5,6-hexa­hydro-1*H*-4,12-meth­ano­benzofuro[3,2-*e*]isoquinolin-7(7a*H*)-one as one of the starting materials.

## Related literature

For general background to methyl­naltrexone (MNTX) bromide and *R*-MNTX, see: Crabtree (1984 ▶); Doshan & Perez (2006 ▶). For the bioactivity and synthesis of *S*-MNTX, see: Wagoner *et al.* (2006 ▶). 
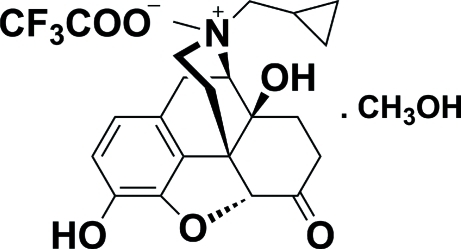


## Experimental

### 

#### Crystal data

C_21_H_26_NO_4_^+^·C_2_F_3_O_2_^−^·CH_4_O
*M* *_r_* = 501.49
Orthorhombic, 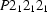

*a* = 9.404 (2) Å
*b* = 12.526 (3) Å
*c* = 19.693 (5) Å
*V* = 2319.8 (10) Å^3^
*Z* = 4
Mo *K*α radiation
μ = 0.12 mm^−1^
*T* = 113 K
0.20 × 0.18 × 0.14 mm

#### Data collection

Rigaku Saturn CCD area detector diffractometer
Absorption correction: multi-scan (*CrystalClear*; Rigaku/MSC, 2005 ▶) *T*_min_ = 0.976, *T*_max_ = 0.983
16033 measured reflections
2336 independent reflections
2261 reflections with *I* > 2σ(*I*)
*R*_int_ = 0.030

#### Refinement

*R*[*F* ^2^ > 2σ(*F* ^2^)] = 0.032
*wR*(*F* ^2^) = 0.079
*S* = 1.07
2336 reflections
349 parameters
66 restraints
H-atom parameters constrained
Δρ_max_ = 0.22 e Å^−3^
Δρ_min_ = −0.19 e Å^−3^

### 

Data collection: *CrystalClear* (Rigaku/MSC, 2005 ▶); cell refinement: *CrystalClear*; data reduction: *CrystalClear*; program(s) used to solve structure: *SHELXS97* (Sheldrick, 2008 ▶); program(s) used to refine structure: *SHELXL97* (Sheldrick, 2008 ▶); molecular graphics: *SHELXTL* (Sheldrick, 2008 ▶); software used to prepare material for publication: *XCIF* in *SHELXTL*.

## Supplementary Material

Crystal structure: contains datablocks I, New_Global_Publ_Block. DOI: 10.1107/S1600536810041164/hg2721sup1.cif

Structure factors: contains datablocks I. DOI: 10.1107/S1600536810041164/hg2721Isup2.hkl

Additional supplementary materials:  crystallographic information; 3D view; checkCIF report

## Figures and Tables

**Table 1:** Hydrogen-bond geometry (Å, °)

*D*—H⋯*A*	*D*—H	H⋯*A*	*D*⋯*A*	*D*—H⋯*A*
O1—H1⋯O5^i^	0.84	1.79	2.622 (2)	170
O4—H4⋯O7^ii^	0.84	1.86	2.685 (2)	169
O7—H7*A*⋯O6^ii^	0.84	1.88	2.698 (2)	166

Symmetry codes: (i) 
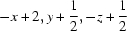
; (ii) 
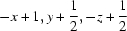
.

**Fig. 1.**
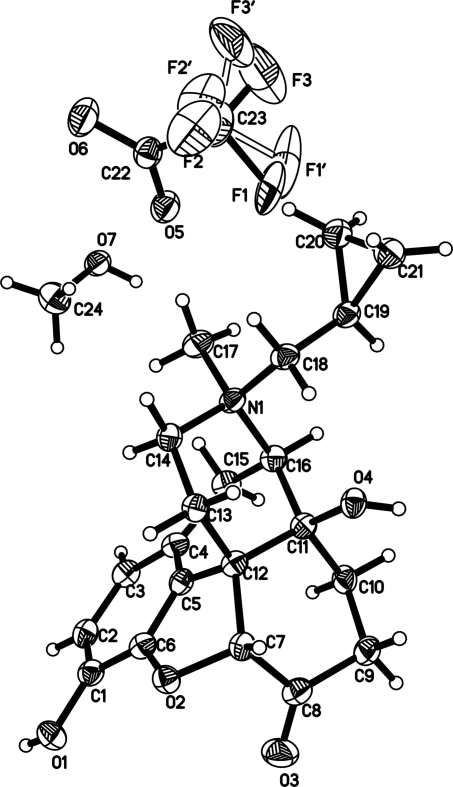
The molecular structure of the title compound, showing 50% displacement ellipsoids.

**Fig. 2.**
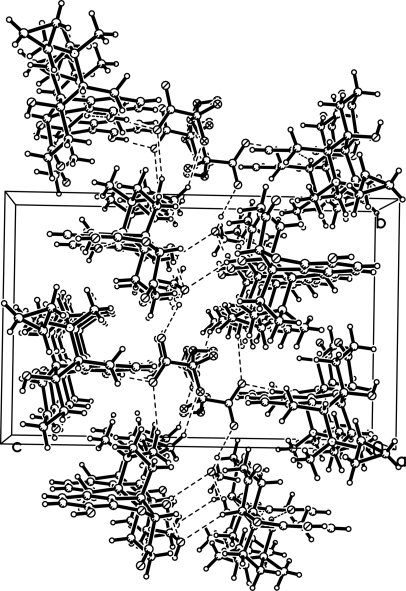
A partial packing diagram of title compound.

## supplementary crystallographic information

### Comment

Methylnaltrexone (MNTX) is a quaternary derivative of the pure opioid antagonist naltrexone, which is a chiral molecule and the quaternary nitrogen can be in *R* or *S* configuration. *R*-MNTX bromide, as a peripheral opioid antagonist, has been used in clinic to refrain from addiction caused by meconium drugs. However, it has been found surprisingly that S-MNTX exhibited opioid agonist activity (Wagoner *et al.*, 2006). In this paper, we report the synthesis and crystal structure of the title compound 2,2,2-trifluoroacetate of *S*-MNTX.

In the crystal structure of the title compound (Fig.1), C_24_H_30_F_3_NO_7_, the conformation of the polycyclic backbone of the noroxymorphone skeleton can be simplified in terms of the angles between the least-squares planes of these rings. Ring A is defined by atoms C1—C6, ring B by atoms C4/C5/C11/C12/C15/C16, ring C by atoms C7—C12, and ring D by atoms C11—C14/ C16/N1. The angle between the rings B and D is 84.5 (6)°. The angle between the planes of ring C and ring A/B/D is respectively 85.8 (6)/80.0 (7)/10.3 (7)°. The structure displays O—H···O hydrogen bonding (Table 1, Fig. 2). The structure exhibits disorder.

### Experimental

A solution of(4R,4aS,7aR,12bS)-3-(cyclopropylmethyl)-4a,9-dihydroxy- 2,3,4,4a,5,6-hexahydro-1H-4,12-methanobenzofuro[3,2-e]isoquinolin-7(7aH) -one(10 g, 29.4 mmol) and MeI(34 g) in 1-methyl-2-pyrrolidinone(100 mL) was stirred at 140 K for 10 h.  The solvent was removed under pressure and the residue was purified by preparative reverse phase column (Waters C18) chromatography using water-methanol-TFA(74.8:25:0.2) as eluent. Two products were isolated. Colorless single crystals of the title compound were obtained from the methanol-water solution.

### Refinement

All H atoms were placed in calculated positions with C—H distances ranging from 0.95 to 1.00 Å and included in the refinement in riding-model approximation with *U*_iso_ = 1.2U_eq_ (1.5U_eq_ for methyl) of the carrier atom. In the absence of significant anomalous scattering effects, Friedel pairs were merged; the absolute configuration was inferred from the synthesis.

### Crystal data

**Table:**

C_21_H_26_NO_4_^+^·C_2_F_3_O_2_^−^·CH_4_O	*F*(000) = 1056
*M**_r_* = 501.49	*D*_x_ = 1.436 Mg m^−^^3^
Orthorhombic, *P*2_1_2_1_2_1_	Mo *K*α radiation, λ = 0.71073 Å
Hall symbol: P 2ac 2ab	Cell parameters from 8884 reflections
*a* = 9.404 (2) Å	θ = 1.6–27.9°
*b* = 12.526 (3) Å	µ = 0.12 mm^−^^1^
*c* = 19.693 (5) Å	*T* = 113 K
*V* = 2319.8 (10) Å^3^	Prism, colorless
*Z* = 4	0.20 × 0.18 × 0.14 mm

### Data collection

**Table:**

Rigaku Saturn CCD area detector diffractometer	2336 independent reflections
Radiation source: fine-focus sealed tube	2261 reflections with *I* > 2σ(*I*)
graphite	*R*_int_ = 0.030
Detector resolution: 14.63 pixels mm^-1^	θ_max_ = 25.0°, θ_min_ = 1.9°
ω and φ scans	*h* = −11→9
Absorption correction: multi-scan *CrystalClear* (Rigaku/MSC, 2005)	*k* = −14→13
*T*_min_ = 0.976, *T*_max_ = 0.983	*l* = −23→23
16033 measured reflections	

### Refinement

**Table:**

Refinement on *F*^2^	Primary atom site location: structure-invariant direct methods
Least-squares matrix: full	Secondary atom site location: difference Fourier map
*R*[*F*^2^ > 2σ(*F*^2^)] = 0.032	Hydrogen site location: inferred from neighbouring sites
*wR*(*F*^2^) = 0.079	H-atom parameters constrained
*S* = 1.07	*w* = 1/[σ^2^(*F*_o_^2^) + (0.051*P*)^2^ + 0.2916*P*] where *P* = (*F*_o_^2^ + 2*F*_c_^2^)/3
2336 reflections	(Δ/σ)_max_ < 0.001
349 parameters	Δρ_max_ = 0.22 e Å^−^^3^
66 restraints	Δρ_min_ = −0.19 e Å^−^^3^

### Special details

**Table:**

Geometry. All esds (except the esd in the dihedral angle between two l.s. planes) are estimated using the full covariance matrix. The cell esds are taken into account individually in the estimation of esds in distances, angles and torsion angles; correlations between esds in cell parameters are only used when they are defined by crystal symmetry. An approximate (isotropic) treatment of cell esds is used for estimating esds involving l.s. planes.
Refinement. Refinement of F^2^ against ALL reflections. The weighted R-factor wR and goodness of fit S are based on F^2^, conventional R-factors R are based on F, with F set to zero for negative F^2^. The threshold expression of F^2^ > 2sigma(F^2^) is used only for calculating R-factors(gt) etc. and is not relevant to the choice of reflections for refinement. R-factors based on F^2^ are statistically about twice as large as those based on F, and R- factors based on ALL data will be even larger. Mo Ka measured Friedel data cannot be used to determine absolute structure in a light atom study.

### Fractional atomic coordinates and isotropic or equivalent isotropic displacement parameters (Å^2^)

**Table:**

	*x*	*y*	*z*	*U*_iso_*/*U*_eq_	Occ. (<1)
O1	1.33431 (18)	0.71301 (14)	0.23591 (8)	0.0281 (4)	
H1	1.3709	0.7168	0.1971	0.042*	
O2	1.14964 (17)	0.71503 (13)	0.35444 (7)	0.0233 (4)	
O3	1.0961 (2)	0.92317 (15)	0.35475 (11)	0.0430 (5)	
O4	0.69083 (18)	0.71936 (12)	0.41814 (7)	0.0239 (4)	
H4	0.6411	0.7735	0.4265	0.036*	
N1	0.6598 (2)	0.54016 (14)	0.31973 (9)	0.0224 (4)	
C1	1.1913 (3)	0.70077 (18)	0.22998 (12)	0.0238 (5)	
C2	1.1185 (3)	0.69242 (18)	0.16778 (12)	0.0258 (6)	
H2	1.1719	0.6938	0.1268	0.031*	
C3	0.9710 (3)	0.68222 (18)	0.16386 (12)	0.0255 (5)	
H3	0.9263	0.6788	0.1206	0.031*	
C4	0.8880 (3)	0.67693 (18)	0.22272 (12)	0.0230 (5)	
C5	0.9618 (3)	0.68362 (17)	0.28299 (11)	0.0215 (5)	
C6	1.1068 (3)	0.69866 (18)	0.28753 (11)	0.0219 (5)	
C7	1.0192 (3)	0.74854 (18)	0.38813 (11)	0.0228 (5)	
H7	1.0236	0.7337	0.4380	0.027*	
C8	0.9985 (3)	0.86850 (19)	0.37436 (12)	0.0271 (6)	
C9	0.8483 (3)	0.90873 (18)	0.38089 (13)	0.0286 (6)	
H9A	0.8448	0.9859	0.3702	0.034*	
H9B	0.8144	0.8987	0.4281	0.034*	
C10	0.7521 (3)	0.84654 (17)	0.33149 (12)	0.0244 (5)	
H10A	0.6544	0.8759	0.3329	0.029*	
H10B	0.7887	0.8539	0.2846	0.029*	
C11	0.7500 (3)	0.72831 (18)	0.35199 (11)	0.0211 (5)	
C12	0.9008 (3)	0.68246 (18)	0.35386 (11)	0.0211 (5)	
C13	0.8942 (3)	0.56643 (18)	0.38041 (12)	0.0222 (5)	
H13A	0.9918	0.5375	0.3844	0.027*	
H13B	0.8503	0.5656	0.4261	0.027*	
C14	0.8082 (3)	0.49697 (17)	0.33281 (12)	0.0240 (5)	
H14A	0.8004	0.4244	0.3525	0.029*	
H14B	0.8592	0.4909	0.2890	0.029*	
C15	0.7269 (3)	0.6745 (2)	0.22605 (12)	0.0258 (6)	
H15A	0.6921	0.6153	0.1971	0.031*	
H15B	0.6896	0.7419	0.2069	0.031*	
C16	0.6658 (3)	0.65996 (17)	0.29849 (11)	0.0225 (5)	
H16	0.5658	0.6871	0.2978	0.027*	
C17	0.5940 (3)	0.4752 (2)	0.26332 (12)	0.0293 (6)	
H17A	0.6626	0.4674	0.2262	0.044*	
H17B	0.5087	0.5115	0.2465	0.044*	
H17C	0.5681	0.4045	0.2806	0.044*	
C18	0.5687 (3)	0.52023 (18)	0.38352 (12)	0.0238 (5)	
H18A	0.5718	0.4431	0.3944	0.029*	
H18B	0.6118	0.5592	0.4221	0.029*	
C19	0.4160 (3)	0.55373 (18)	0.37701 (12)	0.0239 (5)	
H19	0.3997	0.6289	0.3618	0.029*	
C20	0.3038 (3)	0.4746 (2)	0.35699 (13)	0.0328 (6)	
H20A	0.2231	0.5007	0.3293	0.039*	
H20B	0.3345	0.4007	0.3468	0.039*	
C21	0.3133 (3)	0.51176 (19)	0.42924 (12)	0.0307 (6)	
H21A	0.3500	0.4608	0.4634	0.037*	
H21B	0.2386	0.5608	0.4460	0.037*	
F1	0.4670 (15)	0.2811 (8)	0.5019 (7)	0.042 (3)	0.290 (14)
F2	0.475 (2)	0.1198 (14)	0.5336 (8)	0.066 (4)	0.290 (14)
F3	0.3034 (9)	0.1836 (19)	0.4686 (7)	0.089 (4)	0.290 (14)
F1'	0.4075 (11)	0.2763 (4)	0.4989 (4)	0.083 (2)	0.710 (14)
F2'	0.5213 (9)	0.1381 (6)	0.5325 (3)	0.0609 (17)	0.710 (14)
F3'	0.3205 (5)	0.1191 (5)	0.4841 (2)	0.0727 (15)	0.710 (14)
O5	0.5400 (2)	0.24675 (13)	0.38094 (8)	0.0294 (4)	
O6	0.5533 (2)	0.07136 (14)	0.40010 (9)	0.0396 (5)	
C22	0.5196 (3)	0.16372 (19)	0.41422 (11)	0.0256 (5)	
C23	0.4420 (4)	0.1785 (2)	0.48217 (14)	0.0434 (8)	
O7	0.4835 (2)	0.37757 (13)	0.04273 (8)	0.0277 (4)	
H7A	0.4593	0.4383	0.0566	0.042*	
C24	0.6210 (3)	0.3522 (2)	0.06831 (13)	0.0371 (7)	
H24A	0.6853	0.4126	0.0606	0.056*	
H24B	0.6577	0.2890	0.0449	0.056*	
H24C	0.6146	0.3377	0.1171	0.056*	

### Atomic displacement parameters (Å^2^)

**Table:**

	*U*^11^	*U*^22^	*U*^33^	*U*^12^	*U*^13^	*U*^23^
O1	0.0234 (10)	0.0354 (9)	0.0255 (8)	−0.0008 (8)	0.0021 (8)	0.0017 (8)
O2	0.0218 (9)	0.0265 (9)	0.0216 (7)	0.0015 (7)	−0.0026 (7)	0.0008 (7)
O3	0.0314 (11)	0.0279 (10)	0.0698 (14)	−0.0053 (9)	0.0018 (11)	0.0055 (9)
O4	0.0282 (10)	0.0202 (8)	0.0232 (7)	0.0036 (7)	0.0025 (7)	0.0021 (6)
N1	0.0229 (11)	0.0168 (9)	0.0277 (9)	0.0008 (8)	−0.0040 (9)	−0.0001 (8)
C1	0.0260 (14)	0.0194 (11)	0.0261 (12)	0.0008 (10)	−0.0017 (11)	0.0020 (9)
C2	0.0320 (15)	0.0229 (12)	0.0226 (11)	0.0019 (11)	0.0019 (11)	0.0003 (9)
C3	0.0309 (15)	0.0247 (12)	0.0209 (11)	0.0000 (11)	−0.0041 (11)	0.0008 (9)
C4	0.0256 (14)	0.0199 (11)	0.0235 (11)	−0.0009 (10)	−0.0051 (10)	0.0022 (10)
C5	0.0248 (13)	0.0171 (11)	0.0227 (11)	0.0026 (9)	−0.0011 (10)	0.0007 (9)
C6	0.0253 (13)	0.0186 (11)	0.0218 (11)	0.0008 (10)	−0.0042 (10)	0.0020 (9)
C7	0.0249 (13)	0.0220 (12)	0.0217 (10)	0.0016 (10)	−0.0003 (10)	0.0008 (9)
C8	0.0302 (15)	0.0212 (12)	0.0300 (12)	−0.0018 (11)	−0.0025 (11)	−0.0005 (10)
C9	0.0313 (14)	0.0187 (11)	0.0358 (12)	0.0012 (10)	0.0042 (12)	−0.0008 (10)
C10	0.0246 (13)	0.0193 (11)	0.0293 (12)	0.0046 (10)	0.0027 (11)	0.0033 (9)
C11	0.0233 (12)	0.0194 (11)	0.0207 (11)	0.0005 (10)	−0.0003 (10)	0.0027 (9)
C12	0.0237 (13)	0.0189 (11)	0.0206 (10)	−0.0001 (10)	−0.0029 (10)	0.0015 (9)
C13	0.0219 (13)	0.0198 (11)	0.0248 (11)	0.0031 (10)	−0.0021 (10)	0.0041 (9)
C14	0.0223 (13)	0.0183 (11)	0.0312 (12)	0.0040 (10)	−0.0014 (10)	0.0018 (10)
C15	0.0275 (14)	0.0269 (12)	0.0230 (12)	0.0003 (10)	−0.0062 (10)	0.0033 (10)
C16	0.0228 (14)	0.0184 (11)	0.0263 (11)	0.0027 (10)	−0.0034 (11)	0.0045 (9)
C17	0.0321 (15)	0.0276 (12)	0.0283 (12)	−0.0040 (12)	−0.0054 (11)	−0.0035 (10)
C18	0.0257 (14)	0.0180 (11)	0.0276 (11)	−0.0012 (10)	−0.0019 (10)	0.0026 (9)
C19	0.0256 (14)	0.0179 (11)	0.0283 (12)	−0.0001 (10)	−0.0005 (11)	0.0012 (10)
C20	0.0259 (14)	0.0303 (13)	0.0423 (14)	0.0000 (12)	−0.0022 (12)	−0.0062 (11)
C21	0.0289 (15)	0.0276 (13)	0.0356 (13)	0.0007 (11)	0.0013 (12)	0.0049 (10)
F1	0.074 (7)	0.034 (4)	0.018 (3)	0.021 (4)	0.007 (4)	−0.009 (3)
F2	0.113 (9)	0.032 (5)	0.052 (5)	−0.002 (6)	0.045 (5)	0.027 (4)
F3	0.050 (5)	0.135 (9)	0.081 (6)	0.003 (6)	0.019 (4)	−0.017 (6)
F1'	0.148 (6)	0.048 (2)	0.054 (3)	0.054 (3)	0.053 (4)	0.0154 (19)
F2'	0.111 (5)	0.048 (3)	0.0238 (17)	0.001 (3)	0.002 (2)	0.0139 (14)
F3'	0.056 (2)	0.091 (3)	0.071 (2)	−0.001 (2)	0.0386 (18)	0.004 (2)
O5	0.0351 (10)	0.0234 (9)	0.0296 (8)	0.0029 (8)	0.0043 (8)	0.0007 (7)
O6	0.0551 (13)	0.0242 (9)	0.0396 (10)	0.0061 (9)	0.0149 (10)	0.0008 (8)
C22	0.0264 (14)	0.0245 (12)	0.0260 (12)	0.0028 (11)	−0.0020 (11)	−0.0010 (10)
C23	0.060 (2)	0.0361 (16)	0.0343 (15)	0.0108 (15)	0.0087 (15)	−0.0001 (12)
O7	0.0292 (10)	0.0231 (8)	0.0308 (9)	−0.0009 (8)	−0.0008 (8)	−0.0004 (7)
C24	0.0328 (16)	0.0426 (16)	0.0361 (14)	0.0019 (13)	−0.0040 (13)	0.0039 (12)

### Geometric parameters (Å, °)

**Table:**

O1—C1	1.359 (3)	C13—H13B	0.9900
O1—H1	0.8400	C14—H14A	0.9900
O2—C6	1.393 (3)	C14—H14B	0.9900
O2—C7	1.456 (3)	C15—C16	1.549 (3)
O3—C8	1.209 (3)	C15—H15A	0.9900
O4—C11	1.421 (3)	C15—H15B	0.9900
O4—H4	0.8400	C16—H16	1.0000
N1—C17	1.510 (3)	C17—H17A	0.9800
N1—C14	1.519 (3)	C17—H17B	0.9800
N1—C18	1.541 (3)	C17—H17C	0.9800
N1—C16	1.559 (3)	C18—C19	1.501 (3)
C1—C6	1.384 (3)	C18—H18A	0.9900
C1—C2	1.407 (3)	C18—H18B	0.9900
C2—C3	1.395 (4)	C19—C20	1.501 (3)
C2—H2	0.9500	C19—C21	1.506 (3)
C3—C4	1.399 (3)	C19—H19	1.0000
C3—H3	0.9500	C20—C21	1.500 (4)
C4—C5	1.378 (3)	C20—H20A	0.9900
C4—C15	1.517 (4)	C20—H20B	0.9900
C5—C6	1.380 (4)	C21—H21A	0.9900
C5—C12	1.509 (3)	C21—H21B	0.9900
C7—C8	1.539 (3)	F1—C23	1.363 (9)
C7—C12	1.543 (3)	F2—C23	1.290 (10)
C7—H7	1.0000	F3—C23	1.332 (8)
C8—C9	1.505 (4)	F1'—C23	1.309 (6)
C9—C10	1.540 (3)	F2'—C23	1.340 (5)
C9—H9A	0.9900	F3'—C23	1.364 (5)
C9—H9B	0.9900	O5—C22	1.244 (3)
C10—C11	1.535 (3)	O6—C22	1.231 (3)
C10—H10A	0.9900	C22—C23	1.536 (4)
C10—H10B	0.9900	O7—C24	1.423 (3)
C11—C12	1.530 (3)	O7—H7A	0.8400
C11—C16	1.572 (3)	C24—H24A	0.9800
C12—C13	1.546 (3)	C24—H24B	0.9800
C13—C14	1.513 (3)	C24—H24C	0.9800
C13—H13A	0.9900		
			
C1—O1—H1	109.5	C4—C15—H15A	108.7
C6—O2—C7	103.31 (17)	C16—C15—H15A	108.7
C11—O4—H4	109.5	C4—C15—H15B	108.7
C17—N1—C14	108.01 (18)	C16—C15—H15B	108.7
C17—N1—C18	106.54 (18)	H15A—C15—H15B	107.6
C14—N1—C18	108.35 (16)	C15—C16—N1	111.92 (18)
C17—N1—C16	109.65 (17)	C15—C16—C11	111.50 (19)
C14—N1—C16	110.83 (18)	N1—C16—C11	111.26 (17)
C18—N1—C16	113.25 (17)	C15—C16—H16	107.3
O1—C1—C6	120.0 (2)	N1—C16—H16	107.3
O1—C1—C2	124.4 (2)	C11—C16—H16	107.3
C6—C1—C2	115.6 (2)	N1—C17—H17A	109.5
C3—C2—C1	122.6 (2)	N1—C17—H17B	109.5
C3—C2—H2	118.7	H17A—C17—H17B	109.5
C1—C2—H2	118.7	N1—C17—H17C	109.5
C2—C3—C4	120.9 (2)	H17A—C17—H17C	109.5
C2—C3—H3	119.6	H17B—C17—H17C	109.5
C4—C3—H3	119.6	C19—C18—N1	114.64 (19)
C5—C4—C3	115.4 (2)	C19—C18—H18A	108.6
C5—C4—C15	117.9 (2)	N1—C18—H18A	108.6
C3—C4—C15	126.5 (2)	C19—C18—H18B	108.6
C4—C5—C6	124.2 (2)	N1—C18—H18B	108.6
C4—C5—C12	127.2 (2)	H18A—C18—H18B	107.6
C6—C5—C12	108.5 (2)	C20—C19—C18	120.7 (2)
C5—C6—C1	121.1 (2)	C20—C19—C21	59.85 (16)
C5—C6—O2	111.5 (2)	C18—C19—C21	117.2 (2)
C1—C6—O2	127.3 (2)	C20—C19—H19	115.8
O2—C7—C8	107.92 (18)	C18—C19—H19	115.8
O2—C7—C12	104.70 (17)	C21—C19—H19	115.8
C8—C7—C12	110.80 (19)	C21—C20—C19	60.24 (16)
O2—C7—H7	111.1	C21—C20—H20A	117.7
C8—C7—H7	111.1	C19—C20—H20A	117.7
C12—C7—H7	111.1	C21—C20—H20B	117.7
O3—C8—C9	123.4 (2)	C19—C20—H20B	117.7
O3—C8—C7	120.9 (2)	H20A—C20—H20B	114.9
C9—C8—C7	115.5 (2)	C20—C21—C19	59.91 (17)
C8—C9—C10	109.13 (19)	C20—C21—H21A	117.8
C8—C9—H9A	109.9	C19—C21—H21A	117.8
C10—C9—H9A	109.9	C20—C21—H21B	117.8
C8—C9—H9B	109.9	C19—C21—H21B	117.8
C10—C9—H9B	109.9	H21A—C21—H21B	114.9
H9A—C9—H9B	108.3	O6—C22—O5	128.8 (2)
C11—C10—C9	109.23 (19)	O6—C22—C23	115.6 (2)
C11—C10—H10A	109.8	O5—C22—C23	115.5 (2)
C9—C10—H10A	109.8	F2—C23—F1'	113.3 (10)
C11—C10—H10B	109.8	F2—C23—F3	114.8 (10)
C9—C10—H10B	109.8	F1'—C23—F3	76.3 (7)
H10A—C10—H10B	108.3	F2—C23—F2'	21.5 (8)
O4—C11—C12	108.15 (18)	F1'—C23—F2'	107.8 (5)
O4—C11—C10	108.80 (18)	F3—C23—F2'	135.3 (8)
C12—C11—C10	110.9 (2)	F2—C23—F1	105.8 (11)
O4—C11—C16	111.95 (18)	F1'—C23—F1	24.3 (6)
C12—C11—C16	106.19 (18)	F3—C23—F1	100.4 (9)
C10—C11—C16	110.83 (18)	F2'—C23—F1	92.8 (7)
C5—C12—C11	109.05 (19)	F2—C23—F3'	82.5 (8)
C5—C12—C7	97.19 (19)	F1'—C23—F3'	107.2 (4)
C11—C12—C7	118.55 (19)	F3—C23—F3'	38.0 (8)
C5—C12—C13	109.71 (18)	F2'—C23—F3'	103.9 (4)
C11—C12—C13	108.87 (19)	F1—C23—F3'	130.6 (6)
C7—C12—C13	112.68 (19)	F2—C23—C22	120.1 (9)
C14—C13—C12	110.65 (19)	F1'—C23—C22	116.7 (4)
C14—C13—H13A	109.5	F3—C23—C22	107.3 (5)
C12—C13—H13A	109.5	F2'—C23—C22	109.5 (4)
C14—C13—H13B	109.5	F1—C23—C22	106.3 (7)
C12—C13—H13B	109.5	F3'—C23—C22	110.9 (3)
H13A—C13—H13B	108.1	C24—O7—H7A	109.5
C13—C14—N1	113.03 (18)	O7—C24—H24A	109.5
C13—C14—H14A	109.0	O7—C24—H24B	109.5
N1—C14—H14A	109.0	H24A—C24—H24B	109.5
C13—C14—H14B	109.0	O7—C24—H24C	109.5
N1—C14—H14B	109.0	H24A—C24—H24C	109.5
H14A—C14—H14B	107.8	H24B—C24—H24C	109.5
C4—C15—C16	114.4 (2)		
			
O1—C1—C2—C3	−178.6 (2)	C8—C7—C12—C5	79.5 (2)
C6—C1—C2—C3	−0.2 (3)	O2—C7—C12—C11	−152.89 (18)
C1—C2—C3—C4	−1.7 (4)	C8—C7—C12—C11	−36.8 (3)
C2—C3—C4—C5	0.7 (3)	O2—C7—C12—C13	78.3 (2)
C2—C3—C4—C15	175.0 (2)	C8—C7—C12—C13	−165.6 (2)
C3—C4—C5—C6	2.4 (3)	C5—C12—C13—C14	−56.7 (3)
C15—C4—C5—C6	−172.4 (2)	C11—C12—C13—C14	62.5 (2)
C3—C4—C5—C12	178.7 (2)	C7—C12—C13—C14	−163.8 (2)
C15—C4—C5—C12	3.9 (4)	C12—C13—C14—N1	−55.1 (2)
C4—C5—C6—C1	−4.6 (4)	C17—N1—C14—C13	170.77 (19)
C12—C5—C6—C1	178.6 (2)	C18—N1—C14—C13	−74.2 (2)
C4—C5—C6—O2	172.8 (2)	C16—N1—C14—C13	50.6 (2)
C12—C5—C6—O2	−4.0 (3)	C5—C4—C15—C16	−10.2 (3)
O1—C1—C6—C5	−178.3 (2)	C3—C4—C15—C16	175.7 (2)
C2—C1—C6—C5	3.3 (3)	C4—C15—C16—N1	−84.1 (3)
O1—C1—C6—O2	4.7 (4)	C4—C15—C16—C11	41.3 (3)
C2—C1—C6—O2	−173.7 (2)	C17—N1—C16—C15	−47.7 (3)
C7—O2—C6—C5	−20.5 (2)	C14—N1—C16—C15	71.5 (2)
C7—O2—C6—C1	156.8 (2)	C18—N1—C16—C15	−166.53 (19)
C6—O2—C7—C8	−81.9 (2)	C17—N1—C16—C11	−173.2 (2)
C6—O2—C7—C12	36.2 (2)	C14—N1—C16—C11	−54.0 (2)
O2—C7—C8—O3	−17.3 (3)	C18—N1—C16—C11	68.0 (3)
C12—C7—C8—O3	−131.4 (2)	O4—C11—C16—C15	177.34 (18)
O2—C7—C8—C9	157.47 (19)	C12—C11—C16—C15	−64.8 (2)
C12—C7—C8—C9	43.4 (3)	C10—C11—C16—C15	55.7 (3)
O3—C8—C9—C10	116.4 (3)	O4—C11—C16—N1	−56.9 (2)
C7—C8—C9—C10	−58.2 (3)	C12—C11—C16—N1	60.9 (2)
C8—C9—C10—C11	63.6 (2)	C10—C11—C16—N1	−178.62 (19)
C9—C10—C11—O4	62.3 (2)	C17—N1—C18—C19	−61.0 (2)
C9—C10—C11—C12	−56.5 (2)	C14—N1—C18—C19	−176.99 (18)
C9—C10—C11—C16	−174.2 (2)	C16—N1—C18—C19	59.6 (3)
C4—C5—C12—C11	−28.4 (3)	N1—C18—C19—C20	95.2 (2)
C6—C5—C12—C11	148.30 (19)	N1—C18—C19—C21	164.64 (19)
C4—C5—C12—C7	−152.0 (2)	C18—C19—C20—C21	105.7 (3)
C6—C5—C12—C7	24.7 (2)	C18—C19—C21—C20	−111.4 (2)
C4—C5—C12—C13	90.8 (3)	O6—C22—C23—F2	35.2 (10)
C6—C5—C12—C13	−92.5 (2)	O5—C22—C23—F2	−144.7 (10)
O4—C11—C12—C5	175.83 (18)	O6—C22—C23—F1'	178.7 (6)
C10—C11—C12—C5	−65.0 (2)	O5—C22—C23—F1'	−1.3 (7)
C16—C11—C12—C5	55.5 (2)	O6—C22—C23—F3	−98.2 (11)
O4—C11—C12—C7	−74.4 (2)	O5—C22—C23—F3	81.8 (11)
C10—C11—C12—C7	44.8 (3)	O6—C22—C23—F2'	55.9 (5)
C16—C11—C12—C7	165.30 (19)	O5—C22—C23—F2'	−124.1 (4)
O4—C11—C12—C13	56.1 (2)	O6—C22—C23—F1	155.0 (6)
C10—C11—C12—C13	175.36 (17)	O5—C22—C23—F1	−25.0 (7)
C16—C11—C12—C13	−64.2 (2)	O6—C22—C23—F3'	−58.1 (4)
O2—C7—C12—C5	−36.6 (2)	O5—C22—C23—F3'	121.9 (4)

### Hydrogen-bond geometry (Å, °)

**Table:**

*D*—H···*A*	*D*—H	H···*A*	*D*···*A*	*D*—H···*A*
O1—H1···O5^i^	0.84	1.79	2.622 (2)	170
O4—H4···O7^ii^	0.84	1.86	2.685 (2)	169
O7—H7A···O6^ii^	0.84	1.88	2.698 (2)	166

Symmetry codes: (i) −*x*+2, *y*+1/2, −*z*+1/2; (ii) −*x*+1, *y*+1/2, −*z*+1/2.
